# Epidemic history and baseline resistance to NS5A-specific direct acting drugs of hepatitis C virus in Spain

**DOI:** 10.1038/s41598-020-69692-7

**Published:** 2020-08-03

**Authors:** Claudia Palladino, Ifeanyi Jude Ezeonwumelu, Irene Mate-Cano, Pedro Borrego, Paula Martínez-Román, Sonia Arca-Lafuente, Salvador Resino, Nuno Taveira, Verónica Briz

**Affiliations:** 1 Research Institute for Medicines (iMed.ULisboa), Faculty of Pharmacy, Universidade de Lisboa, Av. Prof. Gama Pinto, 1649-003 Lisbon, Portugal; 2 Laboratory of Viral Hepatitis, National Center for Microbiology, Institute of Health Carlos III, Carretera Majadahonda-Pozuelo km 2.2, Majadahonda, 28220 Madrid, Spain; 3 Centro de Investigação Interdisciplinar Egas Moniz (CiiEM), Instituto Universitário Egas Moniz, Caparica, Portugal

**Keywords:** Hepatitis C, Viral infection

## Abstract

Hepatitis C virus (HCV) infection remains a global health problem. Previously, the prevalence of NS5A resistance-associated substitutions (RASs) to elbasvir, a new direct-acting antiviral (DAA) against the NS5A viral protein was assessed by our group before its introduction into clinical use in Spain. However, the origin, epidemic history, transmission dynamics, diversity and baseline RASs to NS5A direct-acting agents of HCV-GT1a in Spain remain unknown. A nationwide cross-sectional survey of individuals chronically-infected with HCV-G1a and DAAs-naïve was performed. HCV population sequencing, phylogenetic analysis and Bayesian methods were used. GT1a clade II was more prevalent than clade I (82.3% vs. 17.7%; *P* < 0.001) and older (estimated origin in 1912 vs. 1952). Clade II epidemic is currently declining whereas clade I epidemic has reached equilibrium. A total of 58 single RASs were identified, which account for the moderate level (10%) of baseline resistance observed. When considering the regional data, marked differences were observed, with thirteen regions showing an intermediate level (5–15%) and one a high level (20%) of resistance. Current HCV-GT1a epidemic in Spain is driven by clade I which seem to have different dissemination routes relative to clade II. A moderate level of baseline RASs to NS5A-DAAs with marked differences among regions was observed. Close surveillance of response to treatment with DAAs will be important.

## Introduction

Hepatitis C virus (HCV) infection is a global health problem with latest data estimates 71.1 million viraemic chronic infections (1.1% of world population)^1^. HCV displays high genetic heterogeneity and is classified into eight major genotypes (1–8)^2^ and 86 subtypes^3^. Worldwide there are significant differences in epidemic history among the HCV genotypes (GT) which may differ in response to treatment, with GT1 being the most represented^4^. Among HCV-infected patients also coinfected with human immunodeficiency virus (HIV) subtype 1a (GT1a) prevails^5–7^.

Despite the high rates of cure with the new direct-acting antivirals (DAAs), the presence of NS5A resistance-associated substitutions (RASs) may compromise the efficacy of NS5A inhibitors. Recently, Zeuzem and colleagues (2017) observed that pretreatment ledipasvir-specific RASs (identified in 8–16% of patients) may compromise treatment outcome particularly in treatment-experienced patients with GT1a, as they found a sustained virological response (SVR) rate of only 76% in patients with pretreatment RASs^8^.

The seroprevalence of HCV infection in Spain is 1.1%^9^ and hepatitis C leads the list of infectious disease related mortality^10^. GT1 accounts for 67% of all HCV infections and GT1a causes 40% of all GT1 infections^11^. Until October 2018 117,452 patients had access to DAAs and 95.5% had SVR in the frame of the national plan of universal access to treatment^12^ while 1.1% of those cured are reinfected^7^.

Our group previously evaluated the prevalence of NS5A RASs to elbasvir before its introduction into clinical use in Spain^13^. However, the HCV-GT1a spatiotemporal distribution, epidemic history and resistance to all NS5A inhibitors in Spain remain unknown. In this study we aimed to make the first description of the origin, epidemic history, transmission dynamics and diversity of HCV-GT1a in Spain.

## Methods

### Study design and patients

Overall, 588 patients harboring HCV-GT1a were included. We used the STROBE checklist^14^ to design and carry out a cross-sectional survey of individuals chronically-infected with HCV-G1a and naïve to NS5A inhibitors who were attended in 84 health centers distributed throughout the national territory (Supplementary Information SI1). The samples were collected prior to anti-NS5A therapy initiation, between October 2014 and October 2015. Genotyping testing was performed using the Real-Time HCV genotype II assay (Abbott Laboratories, Illinois, USA)^15^. The identification of NS5A RASs was performed using plasma specimens at the National Center of Microbiology (*Instituto de Salud Carlos III* [ISCIII]). Anonymized samples and a minimum data set were then transferred to the ISCIII National Biobank (REF: 0000984).

### Amplification and direct sequencing of NS5A gene

Amplification and direct sequencing of complete NS5A gene (1,343 nt) was performed as described elsewhere^13^. Standard Sanger sequencing of the PCR product was carried out using an ABI PRISM 377 DNA sequencer (AppIied Biosystems, USA), with a threshold of sensitivity of 15%^16^.

### HCV subtyping, phylogenetic tree reconstruction and transmission clusters analysis

HCV subtyping was performed using COMET HCV subtyping tool^17^^,^ and HCV-GT1a lineages (clade I and clade II) were confirmed by geno2pheno[HCV] (Bonn, Germany; https://hcv.geno2pheno.org/). The sequences were aligned with the MAFFT algorithm^18^ and manually edited in MEGA v7.0.26^19^. Reference HCV Genotype 1 sequences were retrieved from the Los Alamos HCV database and added to the final alignment (Table SI3). Using the RaxMLGUI v1.5 software, a maximum likelihood tree (ML) was then reconstructed under the GTR + Gamma evolutionary model with 1,000 bootstrap replicates as implemented in RaxMLGUI v1.5^20^. Final visualization and annotation of the ML tree was implemented in iTOL v4.0.3^21^. Transmission clusters including two or more individuals (TC ≥ 2) were identified on the ML tree using ClusterPickerGUI v1.2.3^22^ with bootstrap and genetic distance thresholds set at 70% and 0.045 respectively.

### Bayesian evolutionary analysis

To improve the convergence of MCMC chains during the Bayesian phylogenetic analysis in the BEAST v1.10 package^23^ sequences with < 50% sequence length coverage (n = 14) were excluded from the Spanish HCV-GT1a sequences and the remaining sequences split into two clades, clade I (n = 100) and clade II (n = 474) and analyzed separately. Next, a maximum likelihood tree was built using IqTree^24^ and a temporal signal analysis was performed using Tempest^25^. The Bayesian analysis was carried out as described formerly^26^. Briefly, the SRD06 model of nucleotide substitution^27^^,^ and an uncorrelated lognormal relaxed molecular clock (UCLD) with a Bayesian skyline plot (BSP) coalescent prior were specified in BEAUti in the BEAST package. Because of lack of sufficient temporal signal in our dataset which is often encountered with HCV dataset, and as this can result in unreliable and inaccurate estimates, we relied on previously published data with a strong temporal signal to calibrate the molecular clock^28–30^. Hence, we specified a Normal prior distribution on the clock rate (0.001 ± 0.0005 substitutions/site/year)^31^. For the selection of appropriate molecular clock model, Bayes factor support was calculated by specifying the path-sampling and stepping-stone marginal likelihood estimation (Table SI4)^32^. Three independent Markov Chain Monte Carlo (MCMC) chains were run for 1–2 × 10^8^ generations for clades I and II respectively and combined after discarding 10% burn-in. The convergence of MCMC chains were monitored in Tracer v1.7 (https://tree.bio.ed.ac.uk/software/tracer/). Maximum clade credibility (MCC) trees were summarized using tree annotator and tree visualization was implemented in Figtree v1.4.3 (https://tree.bio.ed.ac.uk/software/figtree/).

### Phylogeography analysis

Discrete phylogenetic analyses of the HCV-GT1a clades I and II epidemic circulating in the autonomous regions in Spain were analyzed using the Bayesian stochastic search variable selection (BSSVS) procedure with an asymmetric model of among-location transition for the determination of the HCV lineage migration rates as implemented in BEAST v1.10 (Table SI4). The MCMC chains were run as described above and the spatiotemporal visualization and estimation of the Bayes factor (BF) support (BF support ≥ 3 was assumed to be relevant^33^ for all the geographical location transitions were performed in the “Spatial Phylogenetic Reconstruction of Evolutionary Dynamics” software using Data-Driven Documents (D3) (spreaD3 v0.9.6)^34^. The georeferencing of the RASs with demonstrated clinical relevance among the Spanish Autonomous Communities (CCAA) was performed by using the GPS coordinates in decimal degrees and the prevalence maps were elaborated using the data visualization tool Tableau 9.3, (Tableau Software, Inc., USA)^35^.

### Resistance-associated substitutions

NS5A-specific clinically relevant RASs analyzed were the following according to the latest European Guidelines: K24G/N/R, K26E, M28A/G/T/V, Q30C/D/E/G/H/I/K/L/N/R/S/T/Y, L31I/F/M/P/V, P32L/S, S38F, H58D/L/R, A92K/T, Y93C/F/H/L/N/R/S/T/W^36^. Fold-change was evaluated according to the European and American Guidelines^36,37^ and additional references listed in Table SI5. RASs were categorized as follow: RASs with a high fold-change (> 100×, > 1,000×, > 10,000× with a probable clinical impact; RASs that have an intermediate impact on efficacy (fold-change < 100×); RASs with a low fold-change (< 20×) with no clinical significant impact^38^. The prevalence of RASs observed regionally was classified as follows: low level (< 5%), intermediate level (5–15%) and high level (> 15%). Codon sites corresponding to these RASs were removed from the HCV NS5A sequence alignment for phylogenetic analysis.

### Statistical analysis

The Pearson chi-square test or Fisher’s exact test were used to analyze categorical variables while continuous variables were compared by Mann–Whitney U test. *P*-values were 2-tailed and statistical significance α was 5%. The SPSS software v.25 (SPSS Inc., Chicago, IL) was used to perform analyses.

### Ethics statement

The study was approved by the Institutional Review Board and the Research Ethic Committee of ISCIII (Nº CEI PI 43_2015) and was conducted in accordance with the Declaration of Helsinki.

## Results

Overall, 588 patients harboring HCV-GT1a were included. The 80.8% of subjects were men and were 50 years old (IQR: 47–53). HCV-monoinfected and HIV/HCV-coinfected patients were equally represented. Clade II was much more prevalent than clade I (82.7% vs. 17.3%; *P* < 0.0001) and clade II was more represented in HCV-monoinfected than in HIV/HCV-coinfected patients (87.0% vs. 78.1%; *P* = 0.004) (Table 1; Figure SI2). Importantly, consistent GT1a lineages results were obtained when comparing the clade assignment by phylogenetic method with the geno2pheno_[HCV]_ algorithm, with only 4 sequences having a different assignment (Table SI6).

**Table 1:** Epidemiological characteristics of enrolled RASs and susceptibility to DAAs.

Characteristics	All patients	HIV status		*P*
		HCV-monoinfected	HIV/HCV-coinfected	
**N, (%)**	588	300 (51.0%)	288 (49.0%)	
**Age, years (median, IQR)**	50 (47; 53)	51 (47; 55)	49 (46; 53)	< 0.001
**Sex, men (n, %)**	475 (80.8)	236 (78.7)	239 (83.0)	0.184
**HCV clade (n, %)**				
I	102 (17.3)	39 (13.0)	63 (21.9)	0.004
II	486 (82.7)	261 (87.0)	225 (78.1)	
**RASs (n, %)**	**N = 58**	**N = 28**	**N = 30**	
K24R	4 (0.7)	2 (0.7)	2 (0.7)	–
M28A/T/V	22 (3.7)	11 (3.7)	11 (3.8)	–
Q30E/H/R	12 (2.0)	7 (2.3)	5 (1.7)	–
L31M	4 (0.7)	1 (0.3)	3 (1.0)	–
H58D	2 (0.3)	–	2 (0.7)	–
Y93C/F/H/N	14 (2.4)	7 (2.3)	7 (2.4)	–
**Double mutations (n, %)**	7 (1.2)	5 (1.7)	2 (0.7)	–
**Patients with reduced susceptibility to DAAs, (n, %)**	50 (8.5)	22 (7.3)	28 (9.7)	0.299
**Resistance to DAAs (n, %)**				
DACLATASVIR	32 (5.4)	12 (4.0)	20 (6.9)	0.116
ELBASVIR	31 (5.3)	11 (3.7)	20 (6.9)	0.075
LEDIPASVIR	35 (6.0)	13 (4.3)	22 (7.6)	0.090
OMBITASVIR	41 (7.0)	20 (6.7)	21 (7.3)	0.766
PIBRENTASVIR	8 (1.4)	5 (1.7)	3 (1.0)	0.725
VELPATASVIR	20 (3.4)	6 (2.0)	14 (4.9)	0.069
1 DAAs	18 (3.1)	10 (3.3)	8 (2.8)	–
2 DAAs	–	–	–	–
3 DAAs	2 (0.3)	1 (0.3)	1 (0.3)	–
4 DAAs	15 (2.6)	6 (2.0)	9 (3.1)	–
5 DAAs	7 (1.2)	–	7 (2.4)	–
6 DAAs	8 (1.4)	5 (1.7)	3 (1.0)	–
Possibly resistant	12 (2.0)	5 (1.7)	7 (2.4)	–

IQR, Interquartile range; DAAs, Direct-Acting Antiviral Agents; RASs: relevant resistance-associated substitutions.

Mann–Whitney U test was used for continuous variables; Pearson Chi-Square test or Fisher’s Exact Test were used for categorical variables. All the tests are 2-sided.

### Male-dominated transmission pairs and clade II viruses predominate the Spanish HCV-GT1a population

**Figure 1:**
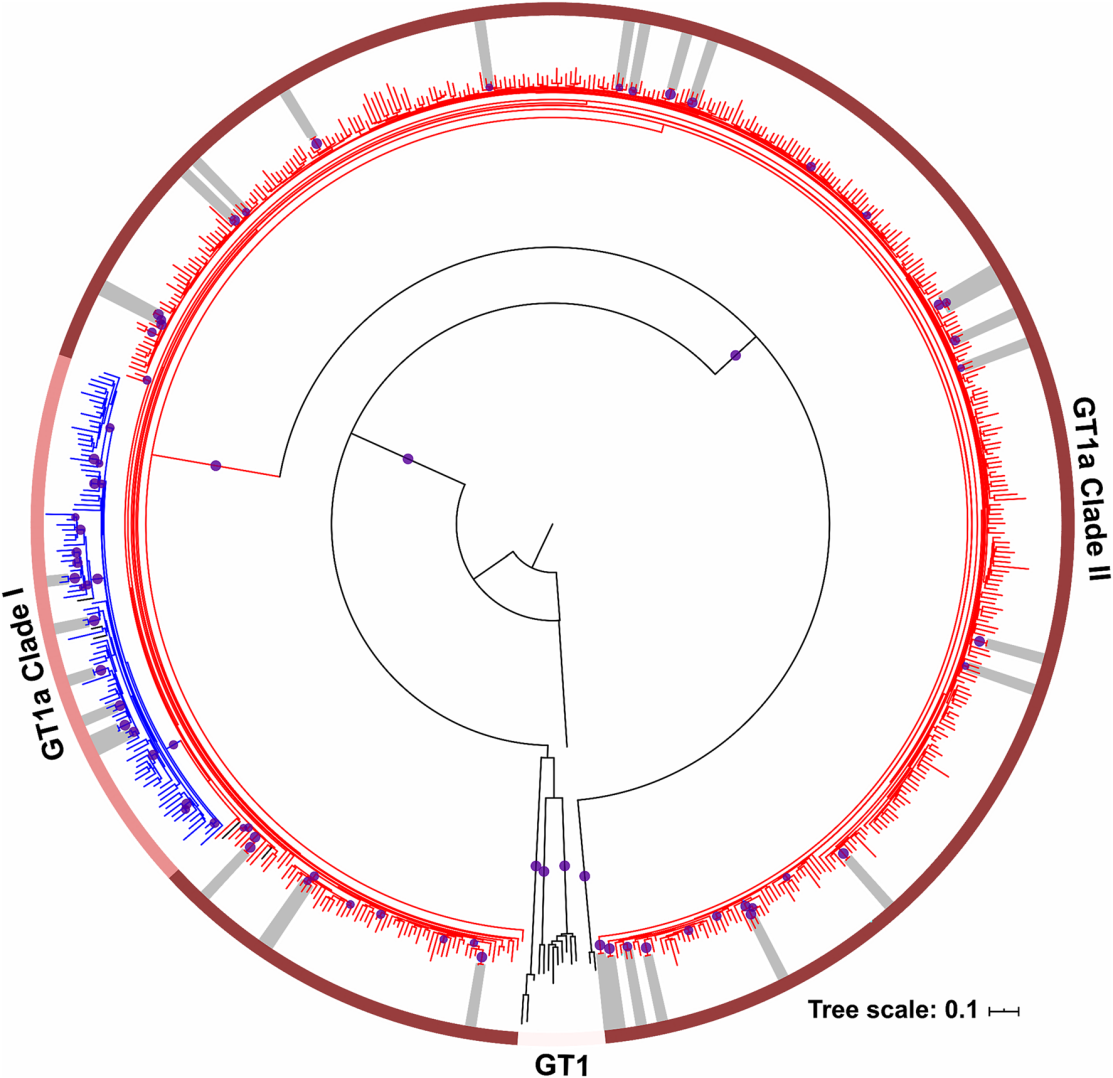
Phylogenetic tree reconstruction of GT1a strains circulating in Spain. A maximum likelihood tree (ML) produced under the GTR + Gamma substitution model with 1,000 bootstrap replicates segregates the GT1a into two clades, clade I (blue) and clade II (red). Bootstrap supports ≥ 70% are depicted as purple filled circles at the nodes and transmission clusters (TC ≥ 2) are highlighted in grey colour.

Twenty-six transmission pairs and three transmission clusters (TC including more than 2 individuals) were identified (70% bootstrap support; genetic distance threshold < 0.045) compared to ten transmission pairs that were identified with a more restrictive transmission cluster criteria (90% bootstrap support; genetic distance threshold < 0.015) (Fig. 1, Table 2), representing 10.5% versus 3.4% (n = 62/588 vs 20/588) of the study population. The transmission pairs and clusters predominantly comprised clade II viruses (79.3%, n = 23/29) and occurred intra-regionally (55.2%, n = 16/29). Fourteen transmission pairs and three clusters (58.6%, n = 17/29) were made up of men only. We further investigated the influence of RASs on the transmission clusters. Identical transmission clusters but short of three transmission pairs (n = 23 vs n = 26) were identified when the RASs sites were not excluded from the alignment; of these, only two clusters, cluster 7 and 8, had transmissible M28V and Q30R + Y93H RASs, respectively (Table 2).

**Table 2:** Characteristics of the identified transmission clusters of GT1a clades I and II strains in Spain.

Cluster	Sample size (number of strains)	Clade	Sex	Age (years)	Region
Cluster 1	2	I; I	F; M	38; 46	ISC; ISC
Cluster 2	2	I; I	F; M	45; 48	GAL; GAL
**Cluster 3**	2	I; I	M; M	43; 45	AND; VAL
Cluster 4	2	I; I	M; M	41; 47	MUR; MUR
**Cluster 5**	2	I; I	M; M	38; 54	MAD; MAD
Cluster 6	2	I; I	M; F	42; 64	GAL; ISC
*Cluster 7	2	II; II	F; F	49; 49	GAL; GAL
**Cluster 8	2	II; II	M; M	49; 52	CAN; PAI
**Cluster 9**	2	II; II	F; M	47; 58	MAD; PAI
Cluster 10	2	II; II	F; M	51; 55	GAL; GAL
**Cluster 11**	2	II; II	M; M	48; 51	ISC; MAD
Cluster 12	2	II; II	F; M	41; 46	CAN; CAN
Cluster 13	2	II; II	F; M	48; 49	CAN; PAI
**Cluster 14**	2	II; II	M; M	44; 46	MUR; GAL
**Cluster 15**	2	II; II	M; M	47; 52	GAL; VAL
Cluster 16	2	II; II	F; M	43; 58	CAS; CAS
Cluster 17	2	II; II	F; F	51; 52	AND; AND
Cluster 18	2	II; II	F; F	46; 52	CAN; CAN
Cluster 19	2	II; II	M; M	43; 48	CAS; CAS
Cluster 20	2	II; II	M; M	49; 54	PAI; PAI
Cluster 21	2	II; II	F; M	50; 51	AND; VAL
**Cluster 22**	2	II; II	M; M	48; 48	AND; NAV
**Cluster 23**	2	II; II	M; M	57; 57	AND; AND
Cluster 24	2	II; II	M; M	42; 42	CAN; MAD
Cluster 25	2	II; II	M; M	47; 48	ARA; ARA
Cluster 26	2	II; II	M; M	46; 55	CAN; AND
Cluster 27	3	II; II; II	M; M; M	42; 47; 48	GAL; GAL; GAL
Cluster 28	3	II; II; II	M; M; M	50; 50; 54	CAN; CAN; CAN
Cluster 29	4	II; II; II; II	M; M; M; M	49; 54; 55; 65	CAN; CAS; PAI; PAI

Clusters are defined by > 70% bootstrap support and < 0.045 genetic distance, and clusters with > 90% bootstrap support and < 0.015 genetic distance are in bold. Clusters with clinically relevant resistance-associated substitutions: *M28V RAS; **Q30R + Y93H.

F, Female; M, Male; autonomous regions of Spain (AND, Andalusia; CAN, Cantabria; CAS, Castile Leon; ISC, Canary Islands; GAL, Galicia; MUR, Murcia; MAD, Madrid; NAV, Navarra; PAI, Basque Country; VAL, Valencia).

The largest transmission cluster, cluster 29 (TC = 4 patients) also had the most diverse population encompassing patients from three different autonomous regions, namely, Cantabria, Castille and Leon and Basque Country. The highest number of transmission pairs and clusters were found in Galicia and Cantabria autonomous regions. Overall these results are consistent with a longstanding HCV epidemic in Spain that is driven by all modes of transmission.

### Origin and spatial-temporal dynamics of GT1a dispersion in Spain

**Figure 2:**
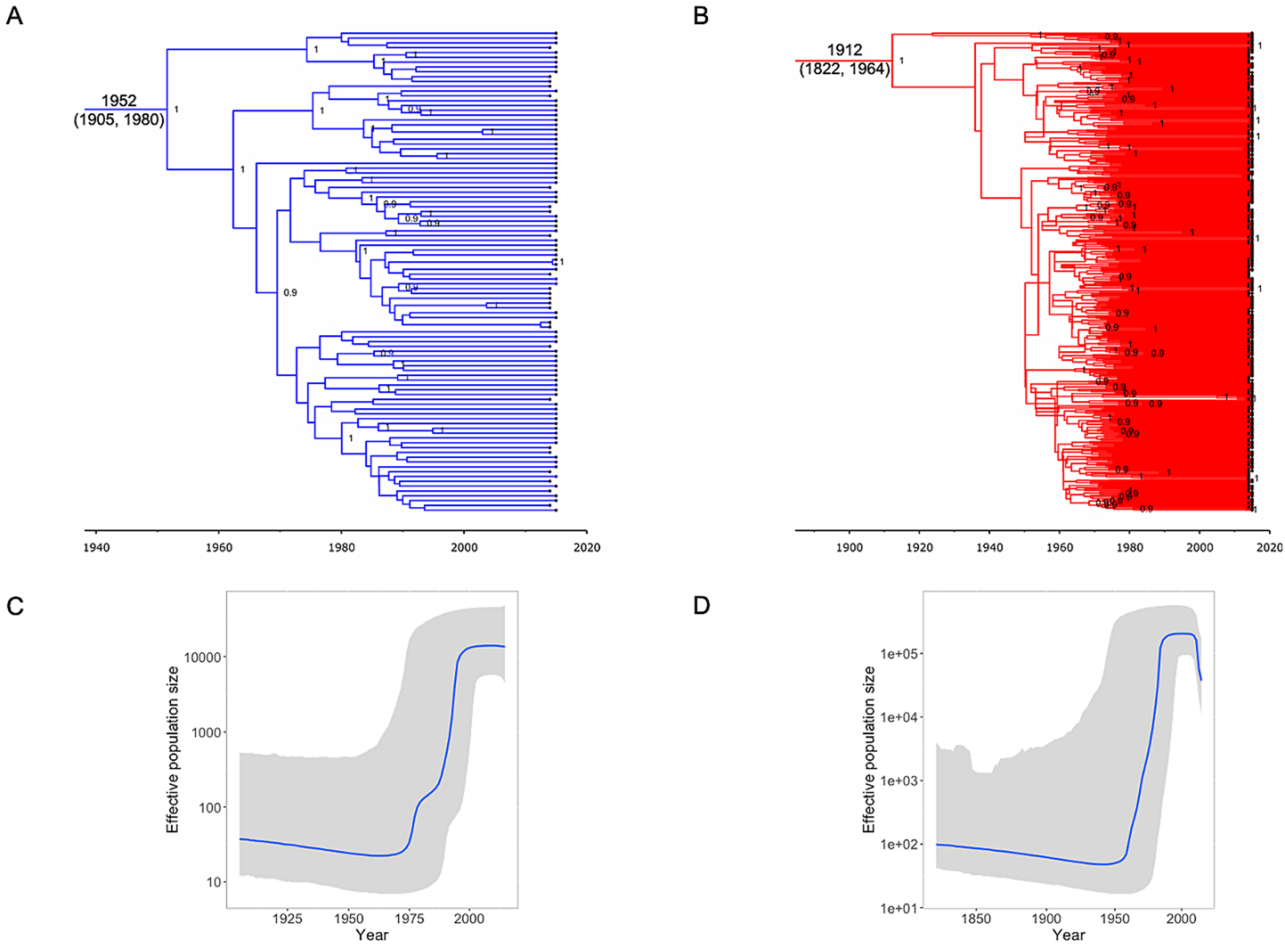
Bayesian estimation of the epidemic history of NS5A GT1a clades in Spain. Maximum clade credibility trees for each of the clades, clade I (**A**) and clade II (**B**) are presented with the mean tMRCA (95% HPD interval) estimates in calendar years annotated at the root nodes for each of the GT1a clades. Posterior probability cut-off value ≥ 0.9 are annotated at the nodes. The Bayesian skyline plots (BSP) for GT1a clade I (**C**) and GT1a clade II (**D**) showing the epidemic growth over time are presented. The solid blue line represents the changes in the median effective population size through time on a log10 scale, with the grey shaded area corresponding to the 95% highest posterior density (95% HPD) interval.

Clade II was introduced in Spain in the beginning of the twentieth century [median estimate of the date of the most recent common ancestor (MRCA), 1912, 95% highest posterior density (95% HPD) interval 1822–1964] whereas clade I was introduced several decades later in 1952 (95% HPD, 1905–1980) (Fig. 2A, B). Both viruses were unnoticed during several decades before going through exponential epidemic growth, clade II starting in the 1950s and clade I twenty years later in the 1970’s (Fig. 2C, D). Clade II epidemic dissemination was more successful than clade I and leveled off in the early 1990’s with N_e_ ≈ 2 × 10^5^ which was almost 20-fold higher than clade I epidemic at its peak (N_e_ ≈ 1.2 × 10^4^ in mid-1990s). However, clade II epidemic is now waning whereas clade I epidemic is still ongoing.

The earliest migration of HCV-GT1a strains in Spain most likely occurred from the Basque country and involved very strongly supported dispersal of clade II viruses from the Basque country to Andalusia and Madrid (BF support = 192.13 and 170.24 respectively) (Table 3). Subsequently, these clade II viruses were successfully propagated from Andalusia and Madrid (BF support = 181.91 and 42.61 respectively). Moreover, Andalusia and Madrid have continued to be the important sources for the dispersal of clade I viruses in contrast to the Basque country which had more support for being a sink (Andalusia-to-Basque country and Galicia-to-Basque country, BF support: > 20 and > 3 respectively) rather than a source of viral dispersion. Thus, the migration pattern of the HCV-GT1a strains in Spain, together with the paraphyly of clade I relative to clade II and its later origin compared to clade II strongly suggest that clade I has evolved from clade II (Fig. 1) and is a driver of the ongoing HCV-GT1a epidemic in Spain.

**Table 3:** Bayes factor and Posterior probabilities for all supported migration events.

Clade I				Clade II			
Migration		Bayes factor^a^	Posterior probability (%)	Migration		Bayes factor^a^	Posterior probability (%)
From	To			From	To		
Andalusia	Balearic Islands	48.06	77.08	Andalusia	La Rioja	181.91	91.78
	Basque Country	20.69	59.14		Extremadura	3.28	16.76
	Galicia	13.51	48.60	Aragon	Catalonia	8.86	35.21
	Asturias	10.08	41.36	Basque Country	Andalusia	192.13	92.18
	Murcia	7.80	35.31		Madrid	170.24	91.26
Asturias	Madrid	32.03	69.14	Catalonia	Aragon	5.34	24.67
	Canary Islands	24.65	63.30	Extremadura	Canary Islands	3.15	16.19
	Andalusia	21.79	60.39	Galicia	Navarra	4.23	20.60
	Galicia	10.81	43.06	La Rioja	Asturias	4.71	22.41
	Castile Leon	9.45	39.80	Madrid	Galicia	42.61	72.34
	Catalonia	3.425	19.33		La Rioja	3.13	16.11
Cantabria	Extremadura	3.18	18.18	Murcia	Castile Leon	15.94	49.45
Catalonia	Extremadura	3.29	18.73	Navarra	Murcia	8.20	33.48
Galicia	Basque Country	3.36	19.05		Galicia	5.99	26.89
Madrid	Catalonia	41.15	74.22				
	Cantabria	29.61	67.44				
	Valencia	5.95	29.38				
	Murcia	5.22	26.75				
	Castile Leon	4.8	25.19				
	Extremadura	4.06	22.10				

^a^Bayes factor (BF): 3 < BF < 10 (support); 10 < BF < 100 (strong support); 100 < BF < 1,000 (very strong support)^33^.

### Baseline polymorphisms and RASs at the NS5A domain

**Figure 3:**
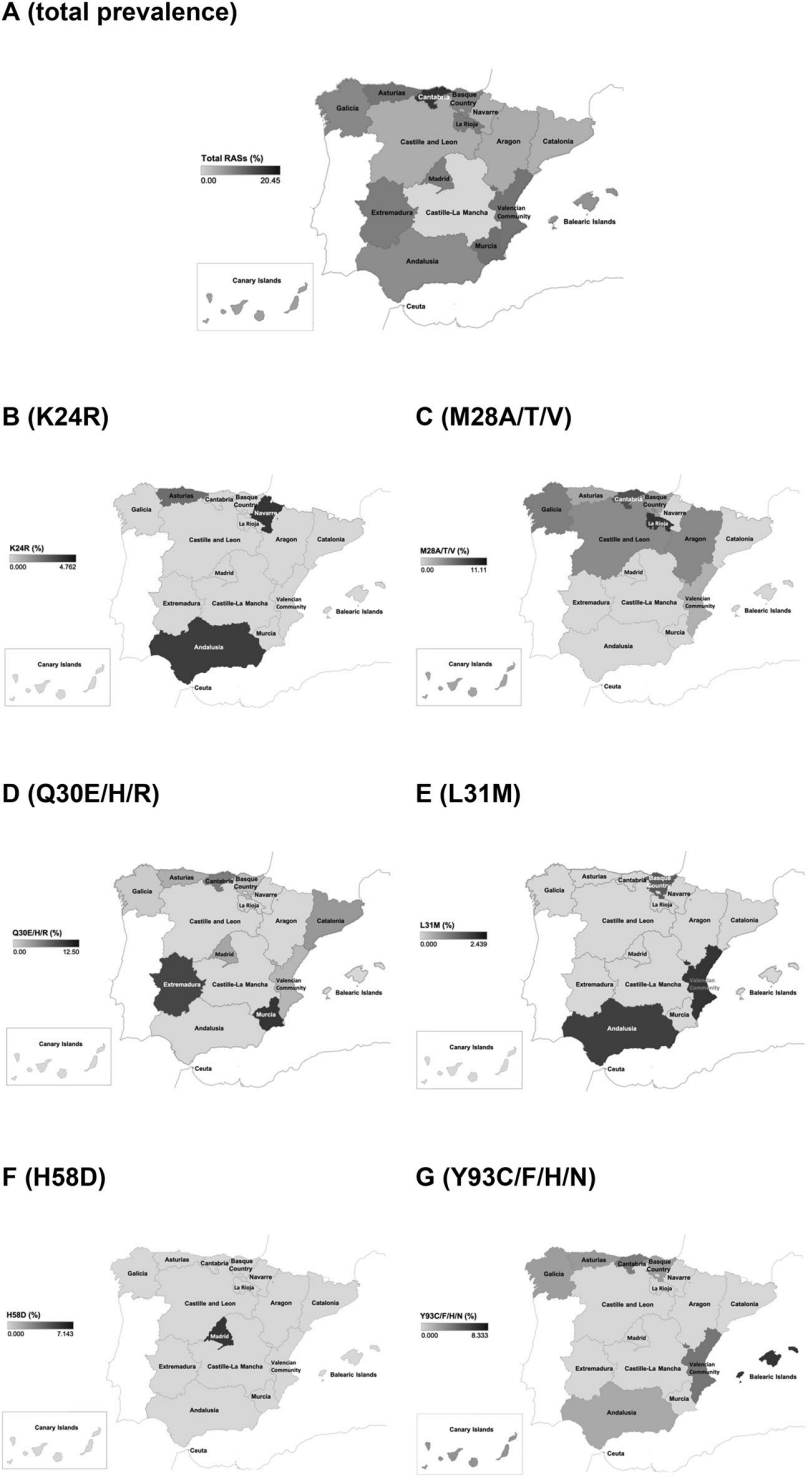
Spatial epidemiology of natural polymorphisms at the HCV *NS5A* gene associated with resistance to NS5A inhibitors among patients infected with HCV genotype 1a in Spain, (**A**) total prevalence of the five resistance mutations under analysis in the 17 Spanish Autonomous Communities and in the city of Ceuta; (**B**) prevalence of K24R (**C**) prevalence of M28A/T/V; (**D**) prevalence of Q30E/H/R; (**E**) prevalence of L31M; (**F**) prevalence of H58D; (**G**) prevalence of Y93C/F/H/N.

Overall, almost 13,000 baseline mutations were detected, of which 8,602 were considered polymorphisms, being found in > 2% of the samples (Supplementary Dataset and Table 4). Viruses bearing RASs were present in 50 individuals (8.5%), 22/300 (7.3%) HCV-monoinfected and 28/288 (9.7%) HCV/HIV-coinfected and were similarly observed in those infected with clade 1 (8.8%; n = 9/102) and clade 2 (8.4%; n = 41/486). Six of those subjects harbored viruses with double RASs and one had triple RASs (14.0%, n = 7/50). In total 58 single RASs were identified in the study population being the most common M28A/T/V (37.9%; n = 22/58), Y93C/F/H/N (24.1%; n = 14/58) and Q30E/H/R (20.7%; n = 12/58). The double mutations 30H + 93H (n = 2), 28V + 30R (n = 2), 30R + 93H (n = 2) and triple mutations 30H + 93H + 24R (n = 1) were also observed (Table 1). The prevalence of RASs observed regionally was as follow: low level (< 5%) in four regions, intermediate (5–15%) in thirteen regions, high (20%) in Cantabria (Fig. 3 and Table 5). Among patients harboring RASs, those with mutations which confer high resistance were: 62.0% (n = 31/50) to daclatasvir, 52.0% (n = 26/50) to ledipasvir, 50.0% (n = 25/50) to ombitasvir, 16.0% (n = 8/50) to elbasvir, 16.0% (n = 8/50) to velpatasvir and 4.0% (n = 2/50) to pibrentasvir; 64.0% (n = 32/50) of patients harbored RASs conferring resistance to more than 1 DAA. Eleven subjects had RASs to velpatasvir and one to pibrentasvir that were not likely to confer a clinically significant impact.

**Table 4:** Polymorphisms and RASs identified in GT1a-infected patients.

	HCV-infected patients						HCV/HIV-coinfected patients					
	Clade I			Clade II			Clade I			Clade II		
A.A position	Mutation	N = 38 patients	%	Mutation	N = 262 patients	%	Mutation	N = 66 patients	%	Mutation	N = 222 patients	%
**24**	**K24Q**	**1**	**0.1**	**K24Q/R**	**3**	**0.1**	**–**	**–**	**–**	**K24R**	**2**	**0.0**
**28**	**M28V**	**1**	**0.1**	**M28V**	**10**	**0.2**	**M28L/T**	**2**	**0.1**	**M28A/T/V**	**10**	**0.2**
**30**	**–**	**–**	**–**	**Q30H/R**	**7**	**0.1**	**–**	**–**	**–**	**Q30E/R**	**5**	**0.1**
**31**	**–**	**–**	**–**	**L31M**	**1**	**0.0**	**L31M**	**2**	**0.1**	**L31M**	**1**	**0.0**
48	R48K/Q	4	0.5	R48H/K/Q	212	3.9	R48K/Q	8	0.5	R48H/K/N/Q/S	187	3.7
**58**	**H58P**	**1**	**0.1**	**H58L/P/Q**	**13**	**0.2**	**H58D/P**	4	0.3	**H58D/P/N/R**	**22**	**0.4**
64	T64A/K/N	20	2.3	T64A/N/S	78	1.4	T64A/N/S	33	2.1	T64A/K/N/S	83	1.6
**92**	**–**	**–**	**–**	**A92P**	**1**	**0.0**	–	–	–	**A92A/D/F/S/V**	**3**	**0.1**
**93**	**Y93C/F**	**3**	**0.4**	**Y93H/N**	**5**	**0.1**	**Y93C**	**3**	**0.2**	**Y93C/H**	**4**	**0.1**
107	K107E/T	20	2.3	T107A/E/K/M	16	0.3	K107E/R/S/T	38	2.4	T107A/K/M	11	0.2
123	R123Q	25	2.9	Q123R	226	4.2	R123Q	38	2.4	Q123R	201	4.0
131	S131T	38	4.4	T131S	1	0.0	S131T	65	4.1	–	–	–
144	I144V	38	4.4	V144I	1	0.0	I144V	64	4.0	V144I	1	0.0
171	E171D	7	0.8	E171D	198	3.6	E171D	15	0.9	E171D	179	3.5
213	A213T	6	0.7	T213A	235	4.3	A213S/T	14	0.9	T213A	207	4.1
226	M226E/L/V	34	4.0	V226E/L/M	132	2.4	M226E/L/V	51	3.2	V226A/E/L/M	153	3.0
293	E293D	1	0.1	D293E/G	219	4.0	E293D/Q	2	0.1	D293E	187	3.7
296	V296I	31	3.6	V296I	21	0.4	V296I	49	3.1	V296I	28	0.6
305	K305R	1	0.1	R305K	237	4.4	K305R	5	0.3	R305K	203	4.0
308	R308G/K	7	0.8	K308N/R	176	3.2	R308K	6	0.4	K308R/S	155	3.1
310	A310P/T	12	1.4	T310A/G/N/P/V	140	2.6	A310E/P/T/V	26	1.6	T310A/G/P/S/V	126	2.5
311	R311A/H/P/Q/S	35	4.1	P311A/Q/R/S/T	16	0.3	R311A/H/K/P/Q/T	60	3.8	P311A/Q	3	0.1
315	V315I	18	2.1	I315C/V	23	0.4	V315F/I	33	2.1	I315F/L/V	34	0.7
326	V326I/L	28	3.3	L326I/M/V	9	0.2	V326I/L	53	3.3	L326I/M/V	14	0.3
348	R348K/Q	22	2.6	R348K/Q	165	3.0	R348K/Q/S/T	44	2.8	R348K/Q	157	3.1
357	–	–	–	R357K	200	3.7	K357R	6	0.4	R357K	193	3.8
368	L368I/V	30	3.5	V368I/L	23	0.4	L368I/V	54	3.4	V368A/I/L/M	26	0.5
392	N392A/D/S	18	2.1	N392A/D/G/H/S	172	3.2	N392D/G/S/T	23	1.4	N392D/G/H/I/S/T/V	165	3.3
400	A400P/S/T/V	7	0.8	S400A/C/D/G/N/P/T/V	219	4.0	A400P/S/T/V	11	0.7	S400A/D/G/P/T	178	3.5
403	G403D/V	4	0.5	A403D/G/I/P/S/T/V	150	2.8	G403A/D/S/V	18	1.1	A403D/G/I/P/T/V	110	2.2
405	P405L/S	6	0.7	P405C/F/H/L/Q/R/S	168	3.1	P405H/L/Q/S	20	1.3	P405C/D/F/H/L/R/S	173	3.4
410	V410A/D/N/T	34	4.0	A410G/V	28	0.5	V410A/D/N	57	3.6	A410T/V	16	0.3
439	G439E	23	2.7	R439D/E/G	226	4.2	G439E	39	2.4	R439E/G	195	3.8
440	A440D	1	0.1	D440A/E/G/T/V	211	3.9	A440D/G/S/T	8	0.5	D440A/G/S/V	175	3.4
441	D441G/S	19	2.2	D441G/N/S	32	0.6	D441G/S	38	2.4	D441E/G	15	0.3
442	T442A	8	0.9	K442A/P/S/T	213	3.9	T442A/K/P/Q	18	1.1	K442A/E/G/N/P/Q/R/S/T	183	3.6
**Total**		**855**	**100.0**		**5.430**	**100.0**		**1.595**	**100.0**		**5.074**	**100.0**

Only polymorphic sites whose frequency is above 2% and RASs are shown; see Table SI6 for the complete dataset of all polymorphisms and mutations. AA, amino acids; resistance-associated substitutions (RASs) are represented in bold and substitution on scored position are underscored.

**Table 5:** Distribution of the RASs polymorphism in HCV-GT1a infected patients throughout the national territory (autonomous communities) of Spain.

Autonomous communities	All RASs			No. of specific RASs						Crude prevalence					
	N. of patients	N. of mutations	Crude prevalence	K24R (N)	M28A/T/V (N)	Q30E/H/R (N)	L31M (N)	H58D (N)	Y93C/F/H/N (N)	K24R (%)	M28A/T/V (%)	Q30E/H/R (%)	L31M (%)	H58D (%)	Y93C/F/H/N (%)
Andalucía	44	4	9.1	2	0	0	1	0	1	4.5	0.0	0.0	2.3	0.0	2.3
Aragon	19	1	5.3	0	1	0	0	0	0	0.0	5.3	0.0	0.0	0.0	0.0
Asturias	33	4	12.1	1	1	1	0	0	1	3.0	3.0	3.0	0.0	0.0	3.0
Cantabria	44	9	20.5	0	4	3	0	0	2	0.0	9.1	6.8	0.0	0.0	4.5
Castile-La Mancha	1	0	0.0	0	0	0	0	0	0	0.0	0.0	0.0	0.0	0.0	0.0
Castile Leon	59	3	5.1	0	3	0	0	0	0	0.0	5.1	0.0	0.0	0.0	0.0
Catalonia	21	1	4.8	0	0	1	0	0	0	0.0	0.0	4.8	0.0	0.0	0.0
Ceuta	1	0	0.0	0	0	0	0	0	0	0.0	0.0	0.0	0.0	0.0	0.0
Extremadura	9	1	11.1	0	0	1	0	0	0	0.0	0.0	11.1	0.0	0.0	0.0
Galicia	102	10	9.8	0	6	1	0	0	3	0.0	5.9	1.0	0.0	0.0	2.9
Balearic Islands	12	1	8.3	0	0	0	0	0	1	0.0	0.0	0.0	0.0	0.0	8.3
Canary Islands	29	2	6.9	0	1	0	0	0	1	0.0	3.4	0.0	0.0	0.0	3.4
La Rioja	9	1	11.1	0	1	0	0	0	0	0.0	11.1	0.0	0.0	0.0	0.0
Madrid	28	3	10.7	0	0	1	0	2	0	0.0	0.0	3.6	0.0	7.1	0.0
Murcia	8	1	12.5	0	0	1	0	0	0	0.0	0.0	12.5	0.0	0.0	0.0
Navarra	21	1	4.8	1	0	0	0	0	0	4.8	0.0	0.0	0.0	0.0	0.0
Basque Country	107	11	10.3	0	4	2	2	0	3	0.0	3.7	1.9	1.9	0.0	2.8
Valencian community	41	5	12.2	0	1	1	1	0	2	0.0	2.4	2.4	2.4	0.0	4.9
**Total**	**588**	**58**	**9.9**	**4**	**22**	**12**	**4**	**2**	**14**	**0.7**	**3.7**	**2.0**	**0.7**	**0.3**	**2.4**

## Discussion

In this study we performed the first nationwide survey assessing the origin, diversity and spatiotemporal transmission dynamics of GT1a using sequences derived from DAA-treatment naïve patients. Phylogenetic analysis confirmed the presence of two divergent GT1a clades, clades I and II^39^. Consistent with our previous observations, clade II showed a prevalence more than fourfold higher than clade I^13,26^. These findings illustrate the distinct geographic distributions of these GT1a clades, since clade I is more common in the United States and both clades are identically represented in other European countries^40,41^. Clade distribution differed among groups of patients, with clade II being more prevalent among HCV-monoinfected patients than among those co-infected with HIV. These differences confirm those reported in our previous nationwide cross-sectional surveys of NS5A RASs to elbasvir^13^ and of NS3 RASs in Spain (2014–2015)^42^, and may be related to differences in mode of transmission of HIV and HCV, such as risky sexual behaviors or parental use of drugs^43,44^.

There were major differences in the origin and spatiotemporal distribution of both clades. The origin of clade II in Spain dates back to the beginning of the twentieth century, preceding clade I by at least 40 years. Clade II transitioned from endemic in the Basque Country to epidemic in Spain. Importantly, the dispersion of clade II from the Basque Country to Andalusia and Madrid coincides with the propagation and spread of clade I from these autonomous communities to the rest of Spain. Overall, Andalusia and Madrid were the most important sources of clade I dissemination in Spain while the Basque Country was the major source of clade II dissemination. De Luca et al.^40^ reported an earlier origin of clade I, 1966 (95% HPD, 1952–1972) compared with clade II, 1975 (95% HPD, 1961–1989) using time-scaled phylogeny applied to NS3 sequences from Italian and German patients and additional sequences from the Los Alamos National Laboratory HCV sequence database with sampling dates from 1977 to 2013 and originated mainly from US but also from other countries such as France and Spain. Overall, these results illustrate fundamental differences in the epidemic behavior of these two GT1a clades both in Spain and in the rest of Europe.

Importantly, our data also shows that clade II epidemic is now declining whereas clade I epidemic has reached equilibrium. The impact of this new clade I epidemic in Spain may be seen on the level of NS3 Q80K prevalence that was reported to be mainly associated with clade I^40–42,45^ and that is associated with reduced treatment response to the macrocyclic protease inhibitor simeprevir^46,47^.

HCV and HIV share common transmission risk behaviors^48^. In Spain, HCV/HIV coinfection has been historically associated with parenteral drug use. Data from the Spanish National Epidemiological Bulleting show that HIV was mainly acquired through parental drug use since the first AIDS cases were reported in 1982^49,50^, while men having sex with men have accounted for most incident cases since the year 2000^51^. Spain is among the eight European countries with high (> 15%) estimates of HCV/HIV coinfection^52^. The most prevalent HCV genotypes associated with parenteral drug use in Europe are GT1a and GT3a. GT1a dominates in Spain among people who inject drugs^53,54^ and HIV/HCV coinfected^7,55^. According to a recent observational analysis, from 1988 to 2015 in North-Eastern Spain there has been an increase in the prevalence of GT1a, GT3 and GT4 associated with male gender and parental use of drugs, currently the most prevalent route of infection, and a concomitant decline of GT1b, associated with transfusion or parenteral/nosocomial transmission^56^. In this context, it is likely that the initial spread of GT1a clade II during the first half of the twentieth century in Spain occurred when HIV had still not found its way to this country, which may clarify the higher prevalence of clade II among the HCV-monoinfected individuals of our cohort.

Regarding the baseline polymorphisms and RAS at the NS5A domain, in a combined analysis carried out in 22 countries of baseline samples from Phase II/III HCV trials, baseline NS5A RASs were present in 13% of 3,501 GT1a samples^8^. Reports of the frequency of naturally occurring NS5A RASs are still scarce in the clinical settings. A cross-sectional cohort of ~ 130 GT1a subjects enrolled in São Paulo (Brazil), reported a prevalence of RASs of 14.6% in HCV-monoinfected (M28V and Q30H/R) and 3.9% in HIV/HCV-coinfected subjects (M28T and Q30H/R)^57^.

A low prevalence of NS5A RASs to the six DAAs evaluated was observed in the present study. RASs were similarly distributed between monoinfected and coinfected patients and between the two GT1a clades in each group. However, when considering the regional data, RASs prevalence level showed a marked geographical variability across regions. In Spain few observational regional or sub-regional studies have been carried out, as resistance testing is only recommended for subjects who are being considered for therapy with grazoprevir/elbasvir. A cohort study including 53 G1a-patients from a main hospital in Madrid (Spain) found that 18.9% (n = 10) harbored at least one RAS^58^. Another study including 166 G1a-patients from a tertiary hospital in A Coruña (Galicia, Spain), found a low (5.5%) prevalence of NS5A RASs using population-based sequencing^59^. Differently from our analyses, this study performed in Galicia did not consider clinically relevant the RASs at position K24.

Thirteen regions showed an intermediate level (5–15%) of baseline RASs to DAAs and Cantabria showed a high level (20%). This suggests that the proportion of patients who will benefit from these treatments may vary according to the Spanish regions. In this sense, it will be important to monitor carefully the response to treatment with NS5A-specific DAAs in patients from these regions and in particular in patients bearing the RASs. The results from these studies will determine whether baseline testing is appropriate for candidates to these new drugs. Nevertheless, results from the observational HCV-TARGET cohort, in USA, indicate that longer treatments may surmount the negative impact of baseline RASs on SVR12 in the clinical setting^60^.

Effective treatment for HCV is currently available in Spain. However, there are several barriers left to disease eradication, including deficiencies in screening and diagnosis. Indeed, almost 50,000 of those living with HCV are still unaware of their infection^61^. Understanding the structural barriers that undermine the implementation of screening programs and the scale-up of diagnosis coverage is fundamental to assure linkage to care and implementation of the HCV care cascade^62,63^.

This study presents some limitations. A more comprehensive dataset of the clinical and epidemiological characteristics of enrolled patient may allow a deeper understanding of the results. Furthermore, the lack of follow-up limited the evaluation of the possible impact of NS5A RASs on the treatment efficacy. Finally, RASs were assessed by population sequencing instead of deep sequencing which may slightly underestimate RASs prevalence^8^.

In conclusion, current HCV-GT1a epidemic in Spain is mainly driven by clade I viruses which seem to have different spreading routes relative to clade II viruses. With the exception of Cantabria, viruses bearing RASs to NS5A-DAAs were present at low to intermediate level in HCV infected patients at baseline. Close surveillance of response to treatment with DAAs will be important.

## Supplementary information

Supplementary file.

Supplementary Datasets.
