# Supplementary material for: Epidemic history and baseline resistance to NS5A-specific direct acting drugs of hepatitis C virus in Spain
Source: Sci Rep. 2020 Aug 3;10:13024. doi: 10.1038/s41598-020-69692-7 (PMC7398927; doi:10.1038/s41598-020-69692-7)
Supplement: Supplementary file 1 — Supplementary file. [file 41598_2020_69692_MOESM1_ESM.pdf]

Type of manuscript: Original

Title: Epidemic history and baseline resistance to NS5A-specific direct acting drugs of Hepatitis C Virus in Spain

Running head: HCV GT1a diversity and drug resistance in Spain

Authors: Claudia Palladino[1*¶], Ifeanyi Jude Ezeonwumelu[1¶], Irene Mate-Cano[2], Pedro Borrego[1], Paula Martínez-Román[2], Sonia Arca de Lafuente[2], Salvador Resino[2], Nuno Taveira[1,3], Verónica Briz[2].

Supplementary Information

SI1. Appendix

Contributing members of the Spanish Group of Chronic Viral Hepatitis that have participated in this epidemiological survey:

1. Área Sanitaria De Ferrol, Ordoñez Barrosa P.;
2. Centro Médico De Asturias, Vilches Vilches R.;
3. Complejo Asistencial Universitario De Burgos, Saez-Royuela Gonzalo F.;
4. Complejo Hospitalario De Navarra, Polo Vigas I.;
5. Complejo Hospitalario De Orense, Esteban Meruendano G.;
6. Complejo Hospitalario De Pontevedra, Trigo Daporta M.;
7. Complejo Hospitalario Universitario De Santiago, Aguilera Guirao A.;
8. Complejo Hospitalario Universitario La Coruña, Cañizares Castellanos M.A.;
9. Complejo Hospitalario Xeral-Calde, Coira Nieto A.;
10. Fundación Hospital Alcorcón, Casas Losada M.L.;
11. Fundación Hospital De Jove, Hidalgo Pérez E.;
12. Fundación Jiménez Díaz-Ute, Fernández Roblas R.;
13. Gerencia D Area De Salud De Badajoz, Llerena Y Zafra, Sanchez Alor G.;
14. Gestión Sanitaria De Mallorca (Gesma), Fernandez Baca Gutierrez Del Álamo V.;
15. Hospital Arnau De Vilanova De Valencia, Giner Duran R.;
16. Hospital Can Misses, Hurtado Fernandez A.;
17. Hospital Central De Asturias, Melon Garcia S.;
18. Hospital Central De La Defensa Gomez Ulla, Mateo Maestre M.;
19. Hospital Clinic I Provincial De Barcelona, Marco Reverte F.;

20. Hospital Clinico San Carlos, Culebras Lopez E.;
21. Hospital Clinico Univers De Salamanca, Gutierrez Zufiaurre M.N.;
22. Hospital Clinico Univers. De Valladolid, Hinojosa Mena Bernal C.;
23. Hospital Clinico Univers.Lozano Blesa, Rubio Calvo C.;
24. Hospital Comarcal De Inca, Saurina Gomila J.;
25. Hospital De Basurto, Hernaez Crespo S.;
26. Hospital De Cabueñes, Otero Guerra L.;
27. Hospital De Cruces, López Soria L.;
28. Hospital De Donostia, Cilla Eguiluz C.G.;
29. Hospital De Especialidades De Jerez De La Frontera, López Prieto M.D.;
30. Hospital De Especialidades De Puerto Real, Jesús De La Calle I.;
31. Hospital De Gran Canaria Dr. Negrín, Pena López M.J.;
32. Hospital De Hellin, Romero Portilla C.;
33. Hospital De La Agencia Valenciana De Salud Vega Baja, Gonzalo Jiménez N.;
34. Hospital De La Línea De La Concepción, Casas Ciria F.J.;
35. Hospital De Móstoles, López Fabal F.;
36. Hospital De Palamós, Guinart Sola N.;
37. Hospital De Poniente, Cabezas Fernandez T.;
38. Hospital Do Meixoeiro, Pérez Castro S.;
39. Hospital Don Benito-Villanueva De La Serena, Valle Valencia A.;
40. Hospital El Bierzo, Raya Fernández C.;
41. Hospital Ernest Lluch Martin, Fortuño Cebamanos B.;
42. Hospital Galdakao-Usansolo, Lopez De Goicoechea San Román M.J.;
43. Hospital Garcia Orcoyen, Barrado Blanco L.;
44. Hospital General De Castellón, Gomila Sard B.;

45. Hospital General De Lanzarote, Copado Carretero R.;
46. Hospital General De Segovia, Elizaga Corrales J.;
47. Hospital General Juan Ramón Jimenez, Saavedra Martin J.;
48. Hospital General Rio Carrión, García Valero T.;
49. Hospital General San Jorge, Torres Sopena L.;
50. Hospital General Universitario De Alicante, Gimeno Gascón A.;
51. Hospital General Universitario De Elche, Ruiz Garcia M.;
52. Hospital General Universitario Gregorio Marañón, Vicente Rangel T.;
53. Hospital General Universitario Reina Sofía, Marín Cervantes A.J.;
54. Hospital Infanta Cristina (Badajoz), Sánchez Silos R.;
55. Hospital Infanta Elena, De La Iglesia Salgado M.;
56. Hospital J.M. Morales Meseguer, Guerrero Gomez C.;
57. Hospital Mateu Orfila, Carbo L.;
58. Hospital Nuestra Señora De Sonsoles, Gomez Del Campo Dechado A.;
59. Hospital Povisa S.A, Martinez Otero M.D.;
60. Hospital Puerta De Hierro (Majadahonda), Portero Azorin F.;
61. Hospital Rafael Mendez, Cascales Alcolea E.;
62. Hospital Reina Sofia (Cordoba), Rodríguez Cantalejo F.;
63. Hospital Reina Sofia (Tudela), García Irure J.J.;
64. Hospital San Agustina (Asturias), Sierra Dorado G.;
65. Hospital San Pedro, Martinez Gil C.;
66. Hospital Santa Maria Nai, Garcia Costa J.;
67. Hospital Txagorritxu, Lezaun Bugui M.J.;
68. Hospital Univers Marques De Valdecilla, Crespo García J.;
69. Hospital Universitario De Canarias, Díaz-Flores Estevez F.;

70. Hospital Universitario De Ceuta, López Barba J.;
71. Hospital Universitario De La Princesa, Cardeñoso Domingo L.;
72. Hospital Universitario Dr. Peset, Alberola Enguidanos J.;
73. Hospital Universitario Insular De Gran Canaria, Santana Rodriguez E.;
74. Hospital Universitario Miguel Servet, Martínez Sapiña A.;
75. Hospital V. Alvarez Buylla, Galarraga Gay M.C.;
76. Hospital Virgen De La Concha, Martinez Gonzalez R.;
77. Hospital Virgen De La Salud (Toledo), Zamarrón Fuertes P.;
78. Hospital Virgen De La Victoria, Clavijo Frutos E.;
79. Hospital Virgen Del Castillo, López Yepes M.L.;
80. Hospital Virgen Del Puerto, García Tejero C.;
81. Hospital Virgen Del Rocío, Merino Díaz L.;
82. Laboratorio De Referencia Del Camp De Tarragona I Terres De L´Ebre, Puerta Martinez M.J.;
83. Laboratorio Referencia Catalunya (El Prat De Llobregat), Salvado Costa M.; Soria Guerrero G.;
84. Laboratorio Brsalud, Aznar Cano E.

Figure SI2. Relative frequency of clade I and II in Spanish hepatitis C virus subtype 1a sequences. A) Relative frequency of clade I and II among the study population; B) Relative frequency of clade I and II among the HIV/HCV-infected and HCV-monoinfected patients.

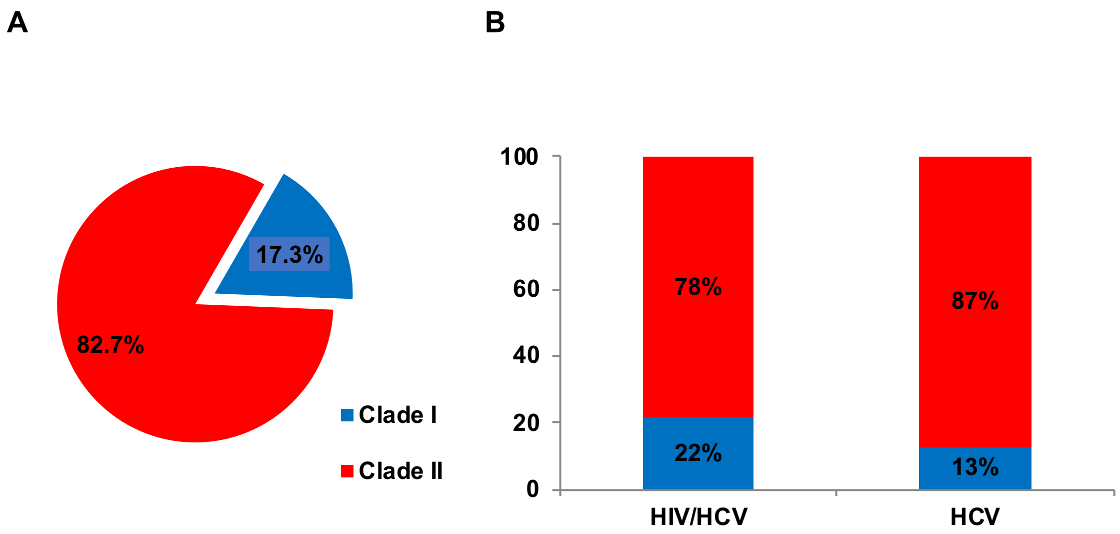

Notes: A) HCV-GT1a clade II was more prevalent than clade I (82.7%, n = 486, vs. 17.3%, n = 102; P<0.0001); B) clade II was more represented in HCV-monoinfected than in HIV/HCV-coinfected patients (87.0%, n = 261, vs. 78.1%, n = 225; P = 0.004).

Table SI3. Sequences used for the phylogenetic analysis.

**Reference HCV-GT1a, accession numbers**	**Sampling date**	**Country of origin/sampling**
AF511950	Not available	Not available
EF407419	Not available	USA
EF407457	Not available	USA
HQ850279	Not available	USA
Other sequences retrieved from the Los Alamos HCV database		
GT1l: KC248197	Not available	Cameroon
KC248196	Not available	UK
KC248193	Not available	Not available
GT1h: KC248199	Not available	Cameroon
KC248198	Not available	Cameroon
GT1g: AM910652	Not available	Spain
GT1e: KC248194	Not available	UK
GT1c: AY051292	Not available	India
GT1b: EU781828	Not available	Not available
EU781827	Not available	Not available
EF032892	2003	Brazil
AY587016	Not available	China

Notes: Reference HCV Genotype 1a with accession numbers: AF511950, EF407419, EF407457, HQ850279 were also retrieved from the Los Alamos HCV database and in

addition to the following reference sequences: GT1l: KC248197, KC248196, KC248193; GT1h: KC248199, KC248198; GT1g: AM910652; GT1e: KC248194; GT1c: AY051292; GT1b: EU781828, EU781827, EF032892, AY587016.

Table SI4: **Bayes Factor estimation for Model comparisons using the Stepping-stone marginal likelihood estimator.** Bayes Factor is given by the ratio of the marginal likelihood estimates (difference between log likelihood of the marginal likelihood estimates): b – a, and d – c.

Molecular Clock model	Bayes factor	BSSVS Substitution Model	Bayes factor
**Clade I**			
a. Strict	39.30	Symmetric	NA
		Asymmetric	
b. UCLD		c. Symmetric	8.68
		d. Asymmetric	
**Clade II**			
a. Strict	526.46	Symmetric	NA
		Asymmetric	
b. UCLD		c. Symmetric	5.21
		d. Asymmetric	

Notes: NA, the relaxed uncorrelated lognormal clock (UCLD) was selected over the Strict clock and was the only model applied to the Bayesian Stochastic Search Variable Selection (BSSVS) substitution models[32].

Table SI5. Studies used to assess the level of resistance of each RAS to each approved NS5A inhibitor (fold-change).

Daclatasvir	1. Fridell RA, Wang C, Sun JH, O'Boyle DR, Nower P, Valera L, et al. Genotypic and phenotypic analysis of variants resistant to hepatitis C virus nonstructural protein 5A replication complex inhibitor BMS-790052 in Humans: In Vitro and In Vivo Correlations. Hepatology. 2011;54:1924–1935.<br>2. Fridell RA, Qiu D, Wang C, Valera L, Gao M. Resistance analysis of the hepatitis C virus NS5A inhibitor BMS-790052 in an in vitro replicon system. Antimicrob. Agents Chemother. 2010;54:3641–3650.<br>3. Wang C, Jia L, O'Boyle DR, Sun JH, Rigat K, Valera L, et al. Comparison of daclatasvir resistance barriers on NS5A from hepatitis C virus genotypes 1 to 6: Implications for cross-genotype activity. Antimicrob. Agents Chemother. 2014;58:5155–5163.<br>4. Fridell RA, Qiu D, Valera L, Wang C, Rose RE, Gao M. Distinct Functions of NS5A in Hepatitis C Virus RNA Replication Uncovered by Studies with the NS5A Inhibitor BMS-790052. J. Virol. 2011;85:7312–7320.<br>5. Wang C, Huang H, Valera L, Sun JH, O'Boyle DR, Nower PT, et al. Hepatitis C virus RNA elimination and development of resistance in replicon cells treated with BMS-790052. Antimicrob. Agents Chemother. 2012;56:1350–1358.<br>6. Bilello JP, Lallos LB, McCarville JF, La Colla M, Serra I, Chapron C, et al. In vitro activity and resistance profile of samatasvir, a novel NS5A replication inhibitor of hepatitis C virus. Antimicrob. Agents Chemother. 2014;58:4431–4442.<br>7. Gao M, Nettles RE, Belema M, Snyder LB, Nguyen VN, Fridell RA, et al. Chemical genetics strategy identifies an HCV NS5A inhibitor with a potent clinical effect. Nature. 2010;465:96–100.

Ledipasvir	8. Zeuzem S, Mizokami M, Pianko S, Mangia A, Han KH, Martin R, et al. NS5A resistance-associated substitutions in patients with genotype 1 hepatitis C virus: Prevalence and effect on treatment outcome. J. Hepatol. [Internet]. 2017;66:910–918. Available from: http://dx.doi.org/10.1016/j.jhep.2017.01.007<br>9. Sarrazin C, Gane EJ, City F, Israel B, Medical D, Hospital AC, et al. Baseline and Post-Baseline Resistance Analyses of Phase 2 / 3 Studies of Ledipasvir / Sofosbuvir ± RBV. 65th Annual Meeting of the American Association for the Study of Liver Diseases Boston, MA Nov 7-11 2014. 2014;<br>10. Wong KA, Worth A, Martin R, Svarovskaia E, Brainard DM, Lawitz E, et al. Characterization of Hepatitis C Virus Resistance from a Multiple-Dose Clinical Trial of the Novel NS5A Inhibitor GS-5885. 2013;57:6333–6340.<br>11. Lawitz EJ, Gruener D, Hill JM, Marbury T, Moorehead L, Mathias A, et al. A phase 1, randomized, placebo-controlled, 3-day, dose-ranging study of GS-5885, an NS5A inhibitor, in patients with genotype 1 hepatitis C. J. Hepatol. 2012;57:24–31.<br>12. European Medicines Agency (EMA). Harvoni - Summary of product characteristics. :1–49.
Ombitasvir	13. Krishnan P, Beyer J, Mistry N, Koev G, Reisch T, DeGoey D, et al. In vitro and in vivo antiviral activity and resistance profile of ombitasvir, an inhibitor of hepatitis C virus NS5A. Antimicrob. Agents Chemother. 2015;<br>14. Degoey DA, Randolph JT, Liu D, Pratt J, Hutchins C, Donner P, et al. Discovery of ABT-267, a pan-genotypic inhibitor of HCV NS5A. J. Med. Chem. 2014;57:2047–2057.<br>15. Wyles DL, Luetkemeyer AF. Understanding Hepatitis C Virus Drug Resistance : Clinical Implications for Current and Future Regimens. Top. Antivir. Med. 2017;25.<br>16. Krishnan P, Tripathi R, Schnell G, Reisch T, Beyer J, Irvin M, et al. Pooled

	analysis of resistance in patients treated with ombitasvir/ABT-450/r and dasabuvir with or without ribavirin in Phase 2 and Phase 3 clinical trials. Hepatology. 2014;60:1134–1135.<br>17. Krishnan P, Tripathi R, Schnell G, Reisch T, Beyer J, Irvin M, et al. Resistance analysis of baseline and treatment-emergent variants in hepatitis C virus genotype 1 in the AVIATOR study with paritaprevir-ritonavir, ombitasvir, and dasabuvir. Antimicrob. Agents Chemother. 2015;59:5445–5454.<br>18. AbbVie. Highlights of Prescribing Information for Viekira Pak [Internet]. 2017. Available from: https://www.rxabbvie.com/pdf/viekirapak_pi.pdf
Elbasvir	19. Black S, Pak I, Ingravallo P, McMonagle P, Chase R, Shaughnessy M, et al. Resistance Analysis of Virologic Failures in Hepatitis C Genotype 1-Infected Patients Treated With Grazoprevir + Elbasvir ± Ribavirin: the C-WORTHY Study [Internet]. Vienna, Austria: 2015. Available from: http://www.natap.org/2015/EASL/EASL_107.htm<br>20. Lahser FC, Bystol K, Curry S, McMonagle P, Xia E, Ingravallo P, et al. The combination of grazoprevir, a hepatitis C virus (HCV) NS3/4A protease inhibitor, and elbasvir, an HCV NS5A inhibitor, demonstrates a high genetic barrier to resistance in HCV genotype 1a replicons. Antimicrob. Agents Chemother. 2016;60:2954–2964.<br>21. Liu R, Curry S, McMonagle P, Yeh WW, Ludmerer SW, Jumes PA, et al. Susceptibilities of genotype 1a, 1b, and 3 hepatitis C virus variants to the NS5A inhibitor elbasvir. Antimicrob. Agents Chemother. 2015;59:6922–6929.<br>22. Merck Sharp & Dohme Corp. ZEPATIER (elbasvir and grazoprevir) PRESCRIBING INFORMATION [Internet]. 2017;Available from: https://www.accessdata.fda.gov/drugsatfda_docs/label/2017/208261s002lbl.pdf
Velpatasvir	23. Lawitz EJ, Dvory-Sobol H, Doehle BP, Worth AS, McNally J, Brainard DM, et al. Clinical resistance to velpatasvir (GS-5816), a novel pan-genotypic inhibitor

	of the hepatitis C virus NS5A protein. Antimicrob. Agents Chemother. 2016;<br>24. Feld JJ, Jacobson IM, Asselah T, Ruane PJ, Gruener N, Abergel A, et al. Sofosbuvir and Velpatasvir for HCV Genotype 1, 2, 4, 5, and 6 Infection. N. Engl. J. Med. 2015;373:2599–2607.<br>25. Curry MP, O'Leary JG, Bzowej N, Muir AJ, Korenblat KM, Fenkel JM, et al. Sofosbuvir and Velpatasvir for HCV in Patients with Decompensated Cirrhosis. N. Engl. J. Med. [Internet]. 2015;151116123036000. Available from: http://www.nejm.org/doi/abs/10.1056/NEJMoa1512614
Pibrentasvir	26. AbbVie. MAVYRET (glecaprevir and pibrentasvir) Highlights of prescribing information. 2018;1–34. Available from: https://www.rxabbvie.com/pdf/mavyret_pi.pdf<br>27. Ng TI, Krishnan P, Pilot-matias T, Kati W, Schnell G, Beyer J, et al. In Vitro Antiviral Activity and Resistance Profile of the Next-Generation Hepatitis C Virus NS5A Inhibitor Pibrentasvir. 2017;61:1–14.<br>28. Krishnan P, Schnell G, Tripathi R, Beyer J, Reisch T, Dekhtyar T, et al. Integrated resistance analysis of CERTAIN-1 and CERTAIN-2 studies in hepatitis C virus-infected patients receiving glecaprevir and pibrentasvir in Japan. Antimicrob. Agents Chemother. 2018;62.

Table SI6. Sequences with inconsistent clade classification based on the phylogenetic analysis and the geno2pheno$_{[HCV]}$

Sequences ID	Clade by phylogenetic analysis	Clade by geno2pheno$_{[HCV]}$
SRT_792	2	1
SRT_698	2	1
SRT_812	2	1
SRT_904	1	2
